# Normal adrenocorticotropic hormone levels do not exclude adrenal insufficiency during immune checkpoint inhibitor therapy: evidence from clinical, steroid, and structural analyses

**DOI:** 10.3389/fendo.2025.1683546

**Published:** 2025-10-20

**Authors:** Yuria Ishibashi, Ryuta Baba, Akira Okada, Yu Otagaki, Takaya Kodama, Gentaro Egusa, Gaku Nagano, Tsuguka Matsuda, Ryoichi Miura, Atsushi Ono, Masataka Tsuge, Noboru Hattori, Haruya Ohno

**Affiliations:** ^1^ Department of Molecular and Internal Medicine, Graduate School of Biomedical and Health Science, Hiroshima University, Hiroshima, Japan; ^2^ Department of Endocrinology and Metabolism, Hiroshima Red Cross Hospital and Atomic Bomb Survivors Hospital, Hiroshima, Japan; ^3^ Department of Gastroenterology, Graduate School of Biomedical and Health Sciences, Hiroshima University, Hiroshima, Japan; ^4^ Liver Center, Hiroshima University Hospital, Hiroshima, Japan

**Keywords:** immune checkpoint inhibitor (ICI), adrenocorticotropic hormone (ACTH), hypophysitis, immune-related adverse events (irAEs), adrenal insufficiency, isolated adrenocorticotropic hormone deficiency

## Abstract

**Introduction:**

Immune checkpoint inhibitor-induced isolated adrenocorticotropic hormone (ACTH) deficiency (ICI-IAD) represents a critical endocrine immune-related adverse event (irAE) that may become life-threatening without timely diagnosis. Most cases present with suppressed ACTH and cortisol levels; however, a subset of patients shows preserved ACTH levels despite biochemical evidence of adrenal insufficiency. The mechanism and clinical implications underlying this discordance remain poorly defined. This study aimed to investigate the pathophysiological basis and clinical significance of preserved ACTH in patients with ICI-IAD.

**Methods:**

This study involved retrospective and prospective analysis of 49 patients diagnosed with ICI-IAD. Based on plasma ACTH levels, patients were categorized into ACTH-preserved (≥10 pg/mL) and ACTH-depleted (<10 pg/mL) groups. Comparisons included clinical characteristics, hormone responses to stimulation tests, steroid metabolite levels, and molecular features of circulating ACTH.

**Results:**

The ACTH-preserved phenotype accounted for 14% of the cohort. Clinical characteristics—including cancer types and ICI regimens—did not differ significantly between the two groups. Despite preserved ACTH levels, cortisol and downstream steroid production remained equally suppressed. All patients who underwent the Synacthen test showed impaired adrenal reserve, confirming that adrenal insufficiency occurred even in cases with preserved-range ACTH values. Corticotropin-releasing hormone stimulation tests revealed similarly blunted pituitary responses in both groups. Gel filtration chromatography identified high-molecular-weight ACTH forms in the preserved group, suggesting altered proopiomelanocortin processing or post-translational modifications that impaired ACTH bioactivity.

**Conclusions:**

Adrenal insufficiency may occur when plasma ACTH levels remain within or above the normal range. Diagnosis based solely on ACTH measurements risks underrecognition of ICI-IAD. Comprehensive endocrine assessment—including dynamic hormone testing and detailed steroid profiling—enhances diagnostic accuracy and informs timely intervention.

## 1 Introduction

The advent of immune checkpoint inhibitors (ICIs) has dramatically transformed the therapeutic landscape for various malignancies (1, 2). By targeting the programmed death-1/programmed death-ligand 1 (PD-1/PD-L1) or cytotoxic T-lymphocyte-associated antigen 4 (CTLA-4) pathways, ICIs enhance antitumor immune responses and have demonstrated unprecedented survival benefits in multiple cancer types, surpassing conventional chemotherapy and molecular targeted agents (3). However, the potent immune activation triggered by ICIs is also associated with immune-related adverse events (irAEs) (4, 5). Among endocrine irAEs, thyroid dysfunction, hypopituitarism, and type 1 diabetes mellitus have been widely reported (6–8). Of particular concern is ICI-induced isolated adrenocorticotropic hormone (ACTH) deficiency (ICI-IAD), a potentially life-threatening condition resulting from its rapid onset and irreversible course unless diagnosed and treated promptly (9).

ICI-IAD typically presents with sustained suppression of ACTH and cortisol levels, reflecting irreversible damage to corticotrophs in the anterior pituitary. In line with current irAE management guidelines, both ACTH and cortisol levels should be measured to assess the clinical status of patients when adrenal insufficiency is suspected (10–12). However, recent reports have documented cases showing transient or even persistent increases in ACTH levels before the onset of adrenal insufficiency (13–15). These findings suggest that structural or functional abnormalities in ACTH—rather than complete depletion—may contribute to ICI-IAD pathogenesis (16). Nonetheless, the mechanisms by which circulating ACTH becomes biologically inactive or undergoes molecular alterations remain poorly understood. Clinical observations at our institution also identified several patients with ICI-IAD who exhibited clear biochemical evidence of adrenal insufficiency despite ACTH concentrations remaining within the reference range. These patients were hypothesized to represent a distinct subgroup characterized by residual but bio-inactive ACTH, along with shared clinical and biochemical features.

This study comprised retrospective and prospective analyses of a cohort of patients diagnosed with ICI-IAD at our institution. Based on baseline ACTH levels at diagnosis, patients were stratified into ACTH-preserved (≥10 pg/mL) and ACTH-depleted (<10 pg/mL) groups, and clinical characteristics, pituitary function, steroidogenic profiles, and gel filtration chromatography (GFC) results were compared between the two groups. The analysis demonstrated that patients with preserved ACTH levels exhibited impaired adrenal steroidogenesis and evidence of structurally altered, high-molecular-weight ACTH as revealed by GFC.

## 2 Materials and methods

### 2.1 Ethics statement

This study was approved by the Institutional Review Board of Hiroshima University (approval number: E2023-0093). All procedures followed the principles outlined in the Declaration of Helsinki. Written informed consent was obtained from all patients enrolled prospectively. For retrospective data, an opt-out consent approach received prior approval from the ethics committee. All samples were anonymized and handled with strict confidentiality. No publication included any patient-identifiable data.

### 2.2 Study design, patient selection, and data source

This single-center, retrospective, and prospective observational study was conducted at the Department of Endocrinology and Diabetes, Hiroshima University Hospital.

**Figure 1:**
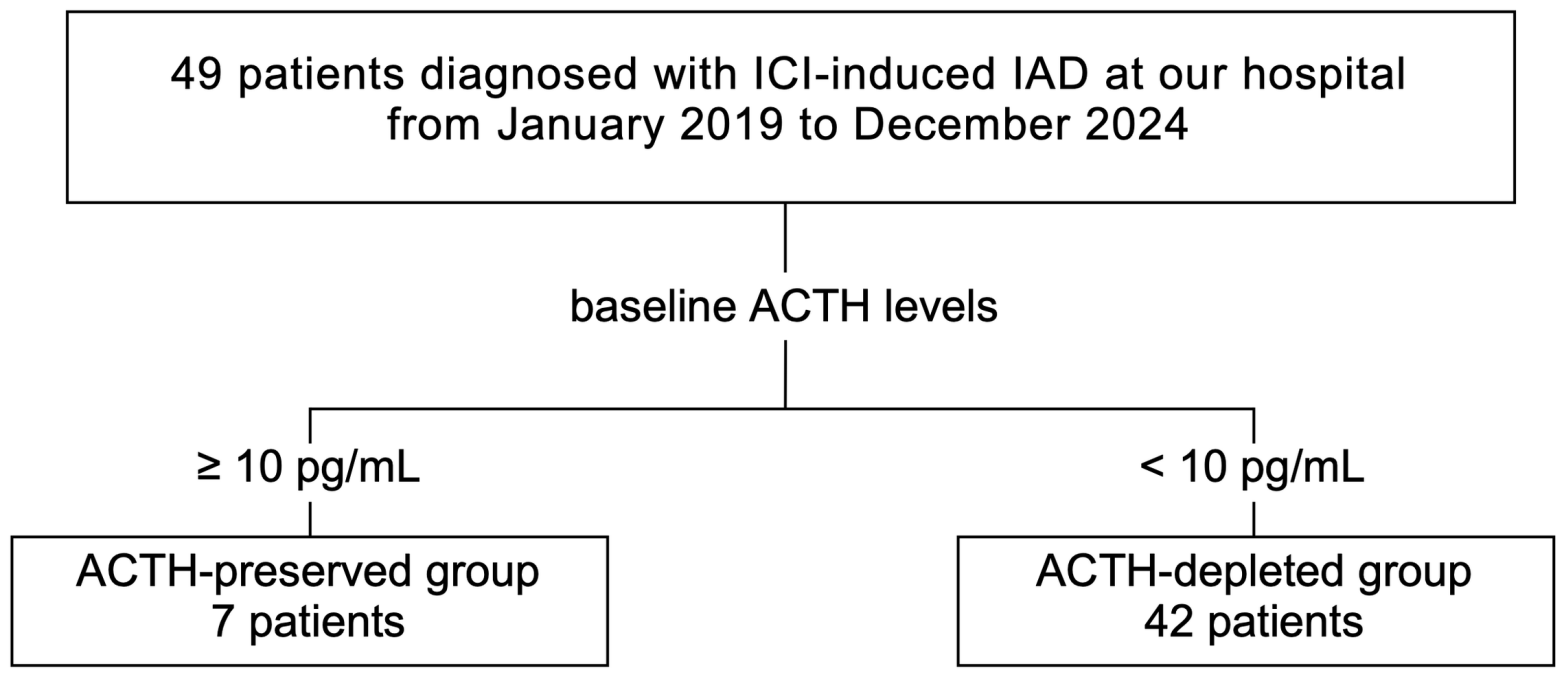
In the combined retrospective and prospective cohorts, 49 patients diagnosed with ICI-IAD between January 2019 and December 2024 were included. Based on baseline plasma ACTH levels, patients were categorized into the ACTH-preserved (≥10 pg/mL, n=7) and ACTH-depleted (<10 pg/mL, n=42) groups. ICI, immune checkpoint inhibitor; ICI-IAD, ICI-induced isolated adrenocorticotropic hormone deficiency; ACTH, adrenocorticotropic hormone.

A total of 49 patients were diagnosed with ICI-IAD between January 2019 and December 2024, including 24 retrospective and 25 prospective cases. Patients were divided into two groups based on baseline plasma ACTH levels at diagnosis: ACTH-preserved (ACTH ≥10 pg/mL) and ACTH-depleted (ACTH <10 pg/mL) groups (**Figure 1**). Clinical characteristics, hormonal responses, and adrenal steroid profiles were compared between these two groups to assess differences in disease phenotypes and pathophysiology. To minimize confounding, patients with pre-existing pituitary disease or primary adrenal insufficiency were excluded. Furthermore, all patients underwent comprehensive pituitary stimulation tests (corticotropin-releasing hormone (CRH), growth hormone-releasing peptide-2 (GHRP2), luteinizing hormone-releasing hormone (LHRH), thyrotropin-releasing hormone (TRH)) and pituitary magnetic resonance imaging (MRI), ensuring that subclinical pituitary disorders or primary adrenal disease were unlikely to have influenced the results. In addition, in a subset of patients, adrenal reserve was directly assessed using the short Synacthen test, which uniformly confirmed impaired adrenal responsiveness. All data were collected from electronic medical records.

Clinical data from patients diagnosed after January 2019 were retrospectively reviewed. In October 2023, the study began a prospective phase following institutional approval. Only patients with confirmed ICI-IAD were included for further analysis. The retrospective cohort (n = 24) primarily provided clinical and hormonal data, whereas the prospective cohort (n = 25) enabled systematic biobanking of serum and plasma samples. Since October 2023, all patients undergoing pituitary function testing at our institution have been prospectively registered, with paired serum and plasma systematically stored together with harmonized clinical information. This design minimized pre-analytical variability and ensured standardized data capture. The prospective collection allowed for advanced analyses, including liquid chromatography-tandem mass spectrometry (LC-MS/MS)-based steroid profiling and GFC. Specimens from 9 prospective patients were consecutively selected, where available, from those with stored samples and used for these assays, in addition to 1 retrospective case previously analyzed for a conference presentation. We compared baseline characteristics and outcomes between retrospective and prospective cohorts and found no significant differences, supporting the validity of pooled analysis (**Supplementary Table S1**). This study design enabled the investigation of ACTH bioactivity and adrenal steroidogenesis in a well-characterized, prospectively followed ICI-IAD cohort.

### 2.3 Diagnosis of ICI-induced hypopituitarism

At Hiroshima University Hospital, the GHRP2 test and the CRH-LHRH-TRH-stimulating test were used to diagnose hypopituitarism. A diagnosis of hypopituitarism was made when hormone deficiencies were detected in any of the anterior pituitary lobes. In the GHRP2 test, growth hormone (GH) deficiency was defined as a peak GH level ≤9 ng/mL measured within 60 min following GHRP2 administration. The CRH-LHRH-TRH stimulation test began in the morning after a 30-min rest period. If the ACTH peak value after the intravenous administration of CRH 100 μg was less than 1.5 times or less than 30 pg/mL, ACTH deficiency was diagnosed. If the peak LH and follicle-stimulating hormone (FSH) levels after intravenous administration of 100 μg LHRH were less than twice the normal levels, and if the peak LH and FSH levels 30 min after LHRH loading were less than twice the normal levels, the patient was diagnosed with LH and FSH deficiencies. If the peak TSH level 30–60 min after intravenous administration of 500 μg TRH was less than 10 μU/mL, the patient was diagnosed with TSH deficiency. Although the participants in this study did not include patients who were using steroid preparations at the time of the examination, three of the ICI-IAD cases had a history of steroid treatment for the purpose of treating malignant tumors.

Adrenal reserve was assessed using the short Synacthen test in 24 of 49 patients. Intravenous ACTH 1–24 (250 µg) was administered, and serum cortisol was measured at baseline and at 30 and 60 min. The peak cortisol was defined as the highest value observed at either 30 or 60 min, and Δcortisol was calculated as peak minus baseline. An insufficient response was defined as a peak cortisol <18 µg/dL. For patients who underwent both Synacthen and CRH stimulation tests, correlations and agreement between pituitary and adrenal responses were analyzed. Agreement between CRH impairment and Synacthen insufficiency was assessed using Cohen’s kappa, and correlations between continuous measures (CRH ratio, Synacthen peak cortisol, and Δcortisol) were evaluated using Spearman’s ρ.

### 2.4 Definition of “preserved” and “depleted” groups

All patients underwent hormonal assessments both at symptom onset and during follow-up. Baseline morning plasma ACTH and serum cortisol concentrations were measured. Patients with plasma ACTH levels ≥10 pg/mL were categorized into the “preserved” group, whereas those with levels <10 pg/mL were placed in the “depleted” group. The 10 pg/mL threshold was selected based on the clinical criteria used to define ACTH suppression in subclinical Cushing’s syndrome (SCS), where ACTH levels below 10 pg/mL typically indicate suppressed endogenous secretion (17, 18). This cut-off provides a clinically relevant approach for patient stratification in this study, despite the fact that it has not been specifically validated for ICI-IAD.

### 2.5 ACTH structure analysis via gel filtration chromatography

GFC was used to analyze ACTH structure and investigate potential differences in molecular size. Plasma samples were collected from 10 patients, including seven in the ACTH-depleted group and three in the ACTH-preserved group. These 10 patients were consecutively selected, where available, from those with stored specimens.

ACTH concentrations in each fraction were measured using the AIA-CL system (AIA-CL2400, Tosoh Corporation, Tokyo, Japan) with the AIA-pack CL ACTH immunoassay reagent (Tosoh Corporation). Chromatographic separation was performed using two tandem TSK gel BioAssist G2SWxl PEEK columns (Tosoh Corporation). The mobile phase comprised 0.05% trifluoroacetic acid (TFA) and 50 mM potassium chloride (KCl), delivered at a flow rate of 0.5 mL/min.

The Prominence high-performance liquid chromatography (HPLC) system (Shimadzu Corporation, Kyoto, Japan), equipped with a CBM-20A system controller and a fraction collector, was employed for sample separation and sequential fraction collection. ACTH immunoreactivity in the collected fractions was analyzed to determine molecular weight profiles.

To enable quantitative comparison of ACTH peaks among patients, the main peak value (representing either high- or normal-molecular-weight ACTH) was corrected using the plasma ACTH concentration measured by the AIA system before chromatography. Specifically, the peak value (measured in arbitrary units) was multiplied by the ratio of the AIA-based ACTH concentration (pg/mL) to the total sum of ACTH values across all chromatographic fractions in the same sample. This correction provided an estimated concentration (pg/mL) for each ACTH peak, allowing for physiologically relevant comparisons across samples.

### 2.6 Steroid analysis

To assess whether preserved ACTH retained biological activity, a comprehensive steroid metabolome analysis was performed using stored serum samples from the same 10 patients described above.

Quantification of pregnenolone, progesterone, cortisol, and other relevant adrenal steroids was conducted using LC-MS/MS. Testing was performed by ASKA Pharmaceutical Medical Co. (Tokyo, Japan). Steroid concentrations were compared between groups to assess the functional effects of ACTH presence or absence.

### 2.7 Statistical analysis

All data were presented as median with interquartile range (IQR). The Mann-Whitney U test and Fisher’s exact test were applied to evaluate statistical significance between groups, as appropriate. Plasma ACTH was measured using the Roche Elecsys system (Roche Diagnostics, Mannheim, Germany; reference range, 7.2–63.3 pg/mL). For statistical analysis, values below the lower limit of detection (1.5 pg/mL) were assigned a value of 1.5 pg/mL. This substitution was applied to both basal ACTH levels and CRH stimulation test results. In contrast, undetectable values in GFC were treated as zero. All statistical analyses were performed using JMP 10 software (SAS Institute Inc.). Statistical significance was set at p < 0.05.

## 3 Results

### 3.1 Clinical characteristics of patients with ICI-IAD

The clinical characteristics and laboratory findings of the ACTH-preserved and ACTH-depleted groups are summarized in **Tables 1** and **2**, respectively.

**Table 1:** Baseline clinical characteristics of 49 patients with ICI-IAD, stratified by ACTH status (ACTH-preserved vs. ACTH-depleted).

Variables	Overall (n=49)	Preserved group (n=7)	Depleted group (n=42)	*P* value
Age at diagnosis, median (IQR)	71 (63–75)	70 (43–74)	71 (65–75)	0.32
Male, n (%)	35 (71.4)	6 (85.7)	29 (69.1)	0.34
Tumor type, n (%)				0.75
Lung	14 (28.6)	3 (42.8)	11 (26.2)	
Liver	9 (18.3)	1 (14.3)	8 (19.0)	
Oral cavity	5 (10.2)	1 (14.3)	4 (9.5)	
Gastric	4 (8.2)	1 (14.3)	3 (7.1)	
Melanoma	4 (8.2)	0 (0)	4 (9.5)	
Pleural mesothelioma	4 (8.2)	0 (0)	4 (9.5)	
Breast	2 (4.1)	0 (0)	2 (4.8)	
Ovary	2 (4.1)	0 (0)	2 (4.8)	
Urothelial	2 (4.1)	0 (0)	2 (4.8)	
Colon	1 (2.0)	1 (14.3)	0 (0)	
Kidney	1 (2.0)	0 (0)	1 (2.4)	
Unknown primary	1 (2.0)	0 (0)	1 (2.4)	
ICI type, n (%)				0.14
Anti PD-1	21 (42.9)	5 (71.4)	16 (38.1)	
Anti PD-L1	9 (18.4)	2 (28.6)	7 (16.7)	
Anti CTLA-4	1 (2.0)	0 (0)	1 (2.4)	
Anti PD-(L)1 + Anti CTLA-4	17 (34.7)	0 (0)	17 (40.4)	
Anti PD-1 + Anti PD-L1	1 (2.0)	0 (0)	1 (2.4)	
Time to develop IAD after starting ICI months, median (IQR)	6.3 (4.2–8.9)	6.0 (5.8–13.2)	6.3 (4.0–8.8)	0.55
Number of ICI administrations until the development of IAD, median (IQR)	5 (3–9)	7 (3–18)	5 (3–9)	0.11
Symptoms at diagnosis, n (%)				
Fatigue	40 (81.6)	5 (71.4)	35 (83.3)	0.45
Anorexia	35 (71.4)	2 (28.6)	33 (78.6)	0.01
Nausea/vomiting	18 (36.7)	1 (14.3)	17 (40.5)	0.18
Hypotension	5 (10.2)	0 (0)	5 (11.9)	0.34
Diarrhea	3 (6.1)	0 (0)	3 (7.1)	0.47
Fever	10 (20.4)	0 (0)	10 (23.8)	0.15
Pituitary MRI, n (%)				0.41
Empty sella	3 (6.1)	0 (0)	3 (7.1)	
Enlargement of the pituitary gland	2 (4.1)	1 (14.3)	1 (2.4)	
No remarkable change	44 (89.8)	6 (85.7)	37 (88.1)	
Other irAEs, n (%)				0.68
Thyroid dysfunction	10 (20.4)	2 (28.6)	8 (19.1)	
Liver	1 (2.0)	0 (0)	1 (2.4)	
None	38 (77.6)	5 (71.4)	33 (78.6)	

*P* values were calculated using the Mann-Whitney U test and Fisher’s exact test, as appropriate.

ACTH, adrenocorticotropic hormone; ICI, immune checkpoint inhibitor; ICI-IAD, ICI-induced isolated ACTH deficiency; MRI, magnetic resonance imaging; irAE, immune-related adverse event.

**Table 2:** Comparison of laboratory findings between the ACTH-preserved and ACTH-depleted groups at diagnosis.

Variables	Overall (n=49)	Preserved group (n=7)	Depleted group (n=42)	*P* value
ACTH (pg/mL)	3.1 (1.5–8.1)	19.5 (12.6–30.5)	2.5 (1.5–5.6)	<0.0001
Cortisol (μg/dL)	0.6 (0.3–1.4)	1.4 (0.4–1.9)	0.55 (0.3–1.33)	0.35
WBC (/μL)	4770 (3650–6075)	4540 (3690–5560)	4820 (3590–6225)	0.43
Eo (/μL)	360 (205–640)	410 (320–640)	330 (200–668)	0.97
Serum sodium (mmol/L)	135 (131–138)	133 (129–139)	135 (131–137)	0.92
Serum potassium (mmol/L)	4 (3.7–4.3)	3.8 (3.7–4.1)	4 (3.7–4.3)	0.49
Serum chloride (mmol/L)	100 (98–104)	103 (97–106)	100 (98–104)	0.46

*P* values were calculated using the Mann-Whitney U test.

ACTH, adrenocorticotropic hormone; ICI, immune checkpoint inhibitor; WBC, white blood cell count; Eo, eosinophils.

#### 3.1.1 Baseline characteristics

Among 49 patients diagnosed with ICI-IAD, seven (14.3%) were classified as ACTH-preserved (ACTH ≥10 pg/mL), and 42 (85.7%) as ACTH-depleted (ACTH <10 pg/mL).

No statistically significant difference in mean age was observed between the preserved and depleted groups (70 [43–74] vs. 71 [65–75] years; p = 0.32), and most patients in both groups were male. Tumor types and ICI regimens did not differ significantly between groups. All patients in the ACTH-preserved group received monotherapy with anti–PD-(L)1 agents, whereas combination therapy with anti–PD-(L)1 and anti–CTLA-4 was exclusively observed in the ACTH-depleted group.

Longer time to IAD onset and a greater number of ICI treatments were observed in the ACTH-preserved group, although the differences did not reach statistical significance (p = 0.55 and p = 0.11, respectively).

Fatigue and anorexia were the most frequently reported symptoms. The ACTH-preserved group exhibited a significantly lower frequency of anorexia (28.6% vs. 78.6%, p = 0.01). Other symptoms and pituitary MRI findings were similar across groups. The incidences of other irAEs, including thyroid dysfunction, appeared comparable.

#### 3.1.2 Hormonal and laboratory findings

Plasma ACTH levels were significantly higher in the ACTH-preserved group (19.5 [12.6 –30.5] pg/mL) compared to the ACTH-depleted group (2.5 [1.5–5.6] pg/mL, P < 0.001). However, serum cortisol levels remained low in both groups (1.4 [0.4–1.9] vs. 0.55 [0.3–1.33] μg/dL, p = 0.35), suggesting the presence of biologically inactive ACTH in the ACTH-preserved group.

White blood cell count, eosinophil count, and serum levels of sodium, potassium, and chloride showed no significant differences between groups.

Sensitivity analyses using alternative ACTH thresholds (5, 7.5, 10, and 15 pg/mL) yielded preserved groups of 18, 13, 7, and 5 patients, respectively. In all scenarios, patients classified as ACTH-preserved still demonstrated impaired adrenal reserve, indicating that our main conclusion—that basal ACTH values alone cannot exclude ICI-IAD—was robust to the choice of cut-off.

### 3.2 Correlation between ACTH and cortisol in preserved vs. depleted groups

**Figure 2:**
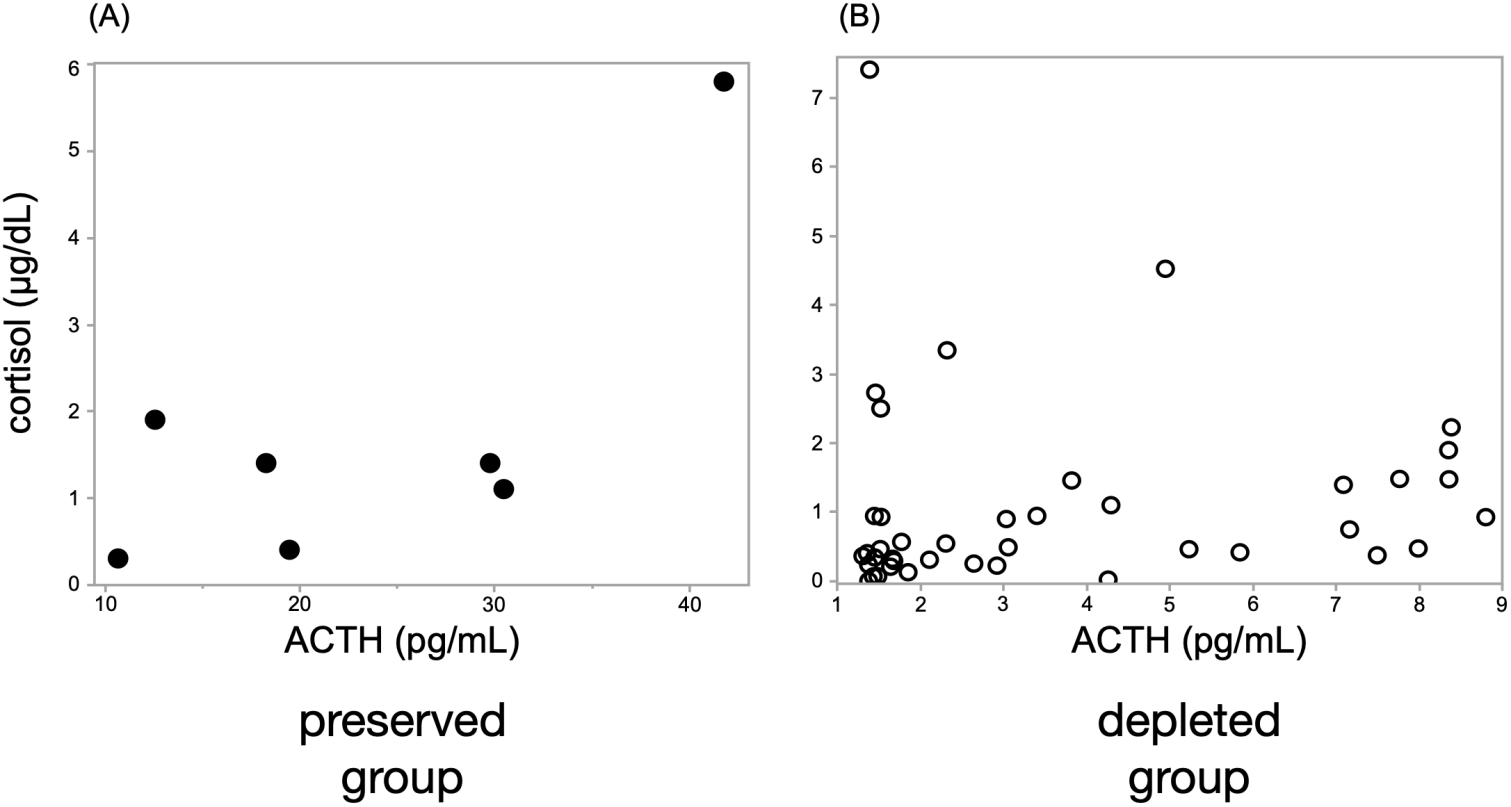
A significant correlation between ACTH and cortisol levels was observed only in the ACTH-depleted group. Scatter plots show Spearman’s rank correlation between ACTH and cortisol levels in **(A)** the ACTH-preserved group (n = 7) and **(B)** the ACTH-depleted group (n = 42). A moderate positive correlation was identified in the ACTH-depleted group (ρ = 0.42, p < 0.01), whereas no significant correlation was found in the preserved group (ρ = 0.43, p = 0.33), suggesting the presence of biologically inactive ACTH in some preserved cases. ACTH, adrenocorticotropic hormone.

The ACTH-preserved group (n = 7) showed no significant correlation between early morning ACTH and cortisol levels (ρ = 0.43, p = 0.33), with several patients exhibiting elevated ACTH levels despite low cortisol concentrations (**Figure 2**). In contrast, the ACTH-depleted group (n = 42) showed a significant positive correlation (ρ = 0.42, p < 0.01), indicating a proportional decrease in ACTH and cortisol levels.

### 3.3 Comparison of ACTH responses to CRH stimulation

**Figure 3:**
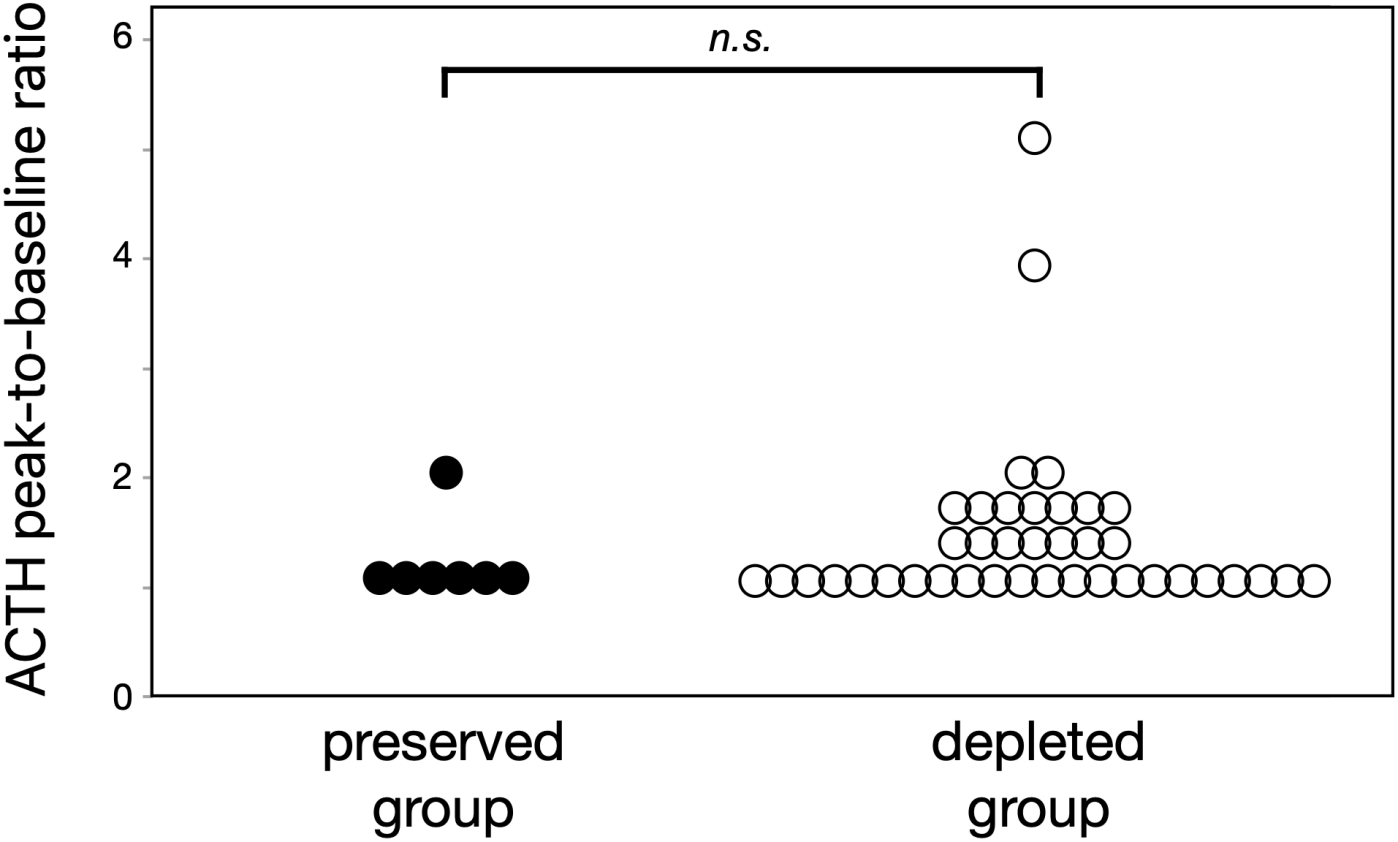
Both ACTH-preserved and ACTH-depleted groups showed similarly blunted responses to CRH stimulation (peak-to-baseline ratio: 1.1 vs. 1.3, p = 0.73), suggesting that ICI-related corticotroph dysfunction is common in ICI-IAD regardless of baseline ACTH levels. These results support the utility of CRH testing when the diagnosis is unclear. ACTH, adrenocorticotropic hormone; CRH, corticotropin-releasing hormone; ICI, immune checkpoint inhibitor; ICI-IAD, ICI-induced isolated ACTH deficiency.

The CRH stimulation test showed an abolished ACTH secretory response in both ACTH-depleted and ACTH-preserved groups (**Figure 3**). The peak ACTH level in both groups remained less than 1.5 times the basal level. No differences emerged in the peak-to-baseline ACTH ratio between the two groups (p = 0.73).

Among 49 patients, the short Synacthen test was performed in 24, comprising 21 in the depleted group and 3 in the preserved group (prospectively enrolled: n = 23; retrospective: n = 1). One retrospective case underwent the Synacthen test, whereas one prospective case did not; both were classified into the ACTH-depleted group. In both groups, peak cortisol levels were markedly impaired, with all patients demonstrating insufficient responses (<18 µg/dL). Median peak cortisol was 4.6 µg/dL (IQR 3.0–6.8) in the depleted group and 11.5 µg/dL (IQR 6.3–13.4) in the preserved group. The corresponding Δcortisol values were 3.9 µg/dL (IQR 2.8–6.8) and 6.7 µg/dL (IQR 4.8–8.4), respectively (**Supplementary Figure S1**). On the Synacthen test, adrenal reserve was uniformly impaired across both the preserved and depleted groups, and no statistical difference could be detected between them.

All patients who underwent the Synacthen test also received CRH stimulation testing, allowing direct comparison between pituitary and adrenal responses. In the 24 patients who underwent both CRH and Synacthen testing, CRH responses were impaired in almost all cases, consistent with the lack of adrenal reserve on Synacthen testing. Correlation analyses showed that the CRH peak-to-basal ratio showed a non-significant correlation with Synacthen peak cortisol (ρ = 0.38, p = 0.07). As all patients demonstrated insufficient Synacthen responses, calculation of kappa coefficients was limited by the absence of discordant cases. These results indicate that pituitary hyporesponsiveness on CRH stimulation generally tracked with impaired adrenal reserve on Synacthen testing.

### 3.4 Clinical characteristics of 10 patients who underwent both LC-MS/MS-based steroid profiling and GFC

**Supplementary Table S2** outlines the clinical characteristics of 10 patients who underwent both LC-MS/MS-based steroid profiling and GFC. Key variables included ACTH group classification (preserved or depleted), sex, age, primary diagnosis, duration from ICI initiation to IAD onset, primary tumor type, ICI regimen, pituitary MRI findings, presence of other endocrine irAEs, and baseline plasma ACTH and serum cortisol concentrations. This information provides essential context for interpreting the differences in steroidogenesis and ACTH molecular structure between the ACTH-preserved (n = 3) and ACTH-depleted (n = 7) groups. Apart from ACTH levels, which served as the basis for classification, no apparent differences in baseline characteristics were identified between the two groups.

### 3.5 Detection of high-molecular-weight ACTH

**Figure 4:**
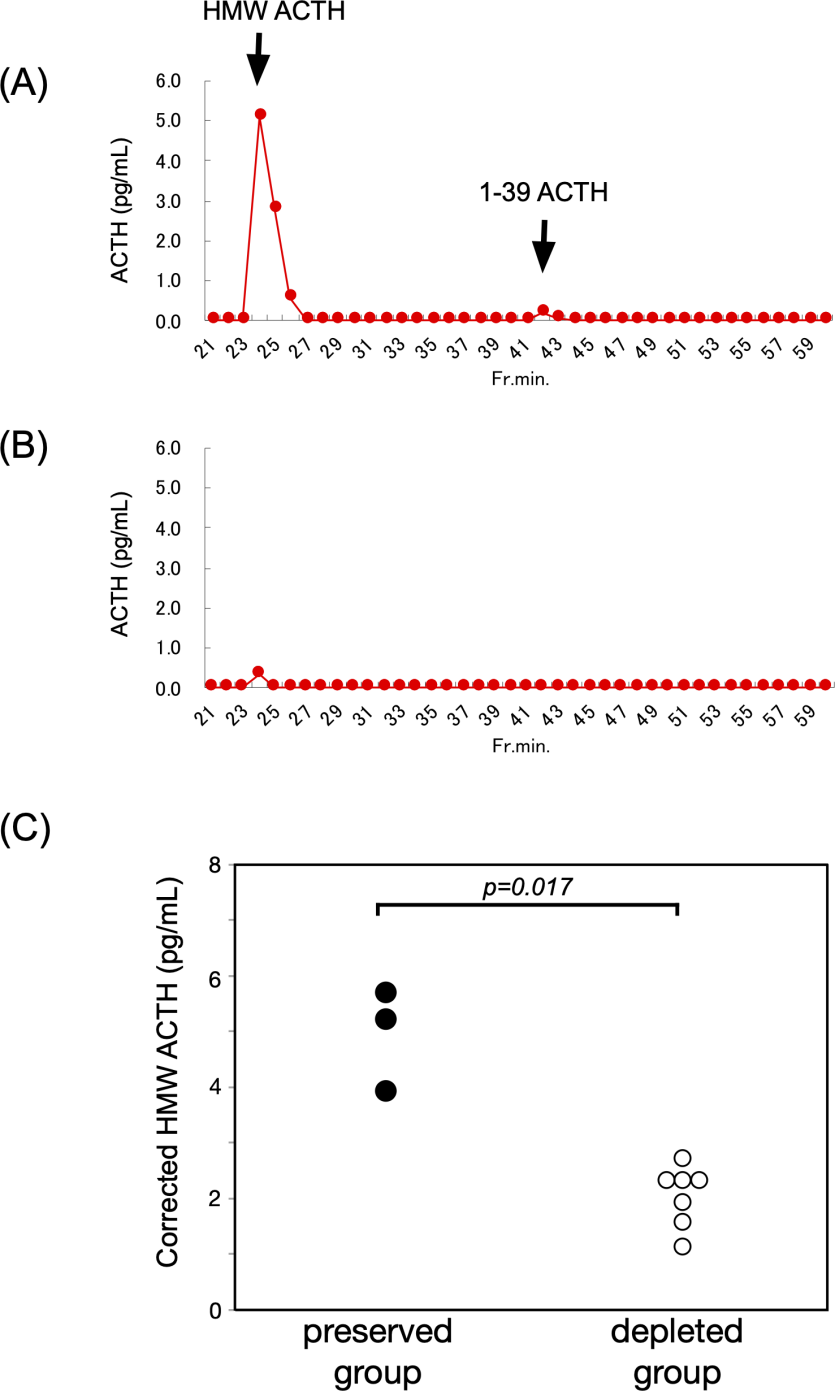
HMW ACTH was prominently detected in the ACTH-preserved group. **(A, B)** Representative GFC profiles from the ACTH-preserved and ACTH-depleted groups, respectively. These profiles illustrate how circulating ACTH molecules are separated according to molecular size. Peaks appearing earlier than the standard ACTH 1–39 correspond to HMW forms, whereas later peaks reflect the normal-sized hormone. This comparison allows visualization of abnormal, larger ACTH species that were selectively present in the preserved group. **(C)** Summary of GFC findings in 10 patients (ACTH-preserved: n = 3; ACTH-depleted: n = 7). Corrected peak values of HMW ACTH were significantly higher in the preserved group compared to the depleted group (median: 5.3 vs. 1.9 pg/mL, p = 0.017). These findings suggest the presence of ACTH with altered molecular structure in the preserved group, potentially due to abnormal POMC processing or post-translational modifications. HMW, high-molecular-weight; ACTH, adrenocorticotropic hormone; GFC, gel filtration chromatography; POMC, proopiomelanocortin.

GFC analysis identified high-molecular-weight ACTH forms in all three ACTH-preserved cases. Elution patterns differed from that of standard ACTH 1–39, with earlier elution peaks indicating the presence of larger molecular species. After correction using plasma ACTH concentrations obtained via the AIA method, the corrected peak ACTH values in the ACTH-preserved group were significantly higher than those observed in the ACTH-depleted group (median: 5.3 vs. 1.9 pg/mL, p = 0.017) (**Figure 4**).

### 3.6 Steroid metabolome findings

**Figure 5:**
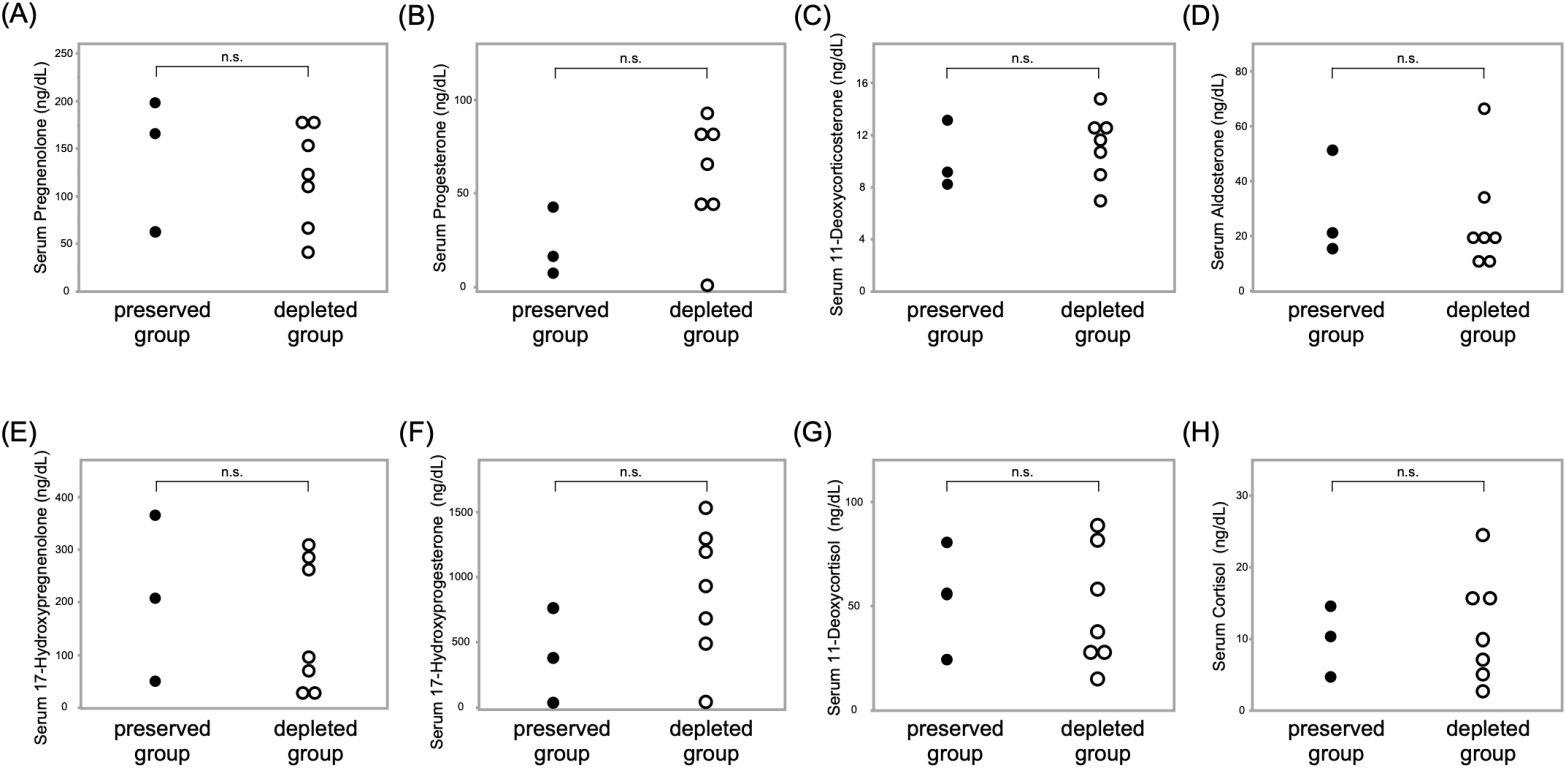
No significant differences in adrenal steroid hormone levels were observed between ACTH-preserved and ACTH-depleted groups in the LC-MS/MS-based steroid profiling Scatter plots show serum concentrations of the following adrenal steroids: **(A)** pregnenolone, **(B)** progesterone, **(C)** 11-deoxycorticosterone, **(D)** aldosterone, **(E)** 17-hydroxypregnenolone, **(F)** 17-hydroxyprogesterone, **(G)** 11-deoxycortisol, and **(H)** cortisol. Each dot represents an individual patient. These findings suggest that adrenal steroidogenesis was comparably suppressed in both groups, regardless of baseline ACTH levels. ACTH, adrenocorticotropic hormone; LC-MS/MS, liquid chromatography–tandem mass spectrometry.

Steroid profiling of 10 patients showed comparable serum adrenal steroid levels between the ACTH-preserved and ACTH-depleted groups. Detailed comparisons are presented in **Figure 5**, demonstrating no statistically significant differences in the levels of pregnenolone (p = 0.65), progesterone (p = 0.17), 11-deoxycorticosterone (p = 0.65), aldosterone (p = 0.65), 17-hydroxypregnenolone (p = 0.65), 17-hydroxyprogesterone (p = 0.17), 11-deoxycortisol (p = 1.0), or cortisol (p = 0.82) between the two groups. The lack of adrenal hormone preservation despite measurable ACTH levels supports the hypothesis that circulating ACTH in the preserved group lacked biological activity.

## 4 Discussion

This study examined ACTH secretion in patients diagnosed with ICI-IAD. A key observation identified 14% of patients (seven out of 49) exhibiting plasma ACTH levels within the normal range despite definitive biochemical evidence of adrenal insufficiency. Clinical and laboratory characteristics outlined in **Tables 1** and **2** showed no significant differences between the ACTH-preserved and ACTH-depleted groups, apart from baseline ACTH levels and the frequency of appetite loss. Furthermore, the distribution of ICI regimens or primary tumor types showed no apparent group-specific patterns. These observations suggest that ACTH preservation or depletion may represent a common manifestation of ICI-IAD, independent of therapeutic agent or tumor origin. Notably, all patients in the ACTH-preserved group had received either anti–PD-1 or anti–PD-L1 monotherapy, with no cases involving combination therapy with anti–CTLA-4. Given that anti–CTLA-4 agents have been more strongly associated with hypophysitis than anti–PD-(L)1 agents, this observation may reflect differences in the underlying mechanisms of pituitary toxicity across ICI subclasses (19). Other potential contributors to adrenal insufficiency, such as prior glucocorticoid exposure, concomitant therapies, or critical illness, were also considered. Notably, patients with primary adrenal disease or known pituitary disorders were excluded, and none were receiving systemic glucocorticoids at the time of diagnosis. Although unrecognized pituitary or adrenal conditions cannot be completely ruled out, the use of dynamic stimulation tests and pituitary MRI substantially reduced the likelihood of such confounding. Although three patients had previously received glucocorticoids for cancer treatment, this exposure was remote and unrelated to the timing of adrenal insufficiency onset. These considerations reinforce that the clinical and biochemical features observed in this study are most consistent with an ICI-induced pathophysiology.

Our correlation analysis revealed contrasting ACTH–cortisol relationships between ACTH-preserved and ACTH-depleted groups. In the ACTH-preserved group, ACTH levels remained within the normal range despite low cortisol concentrations, resulting in a non-significant correlation. This pattern suggests that ACTH secretion is quantitatively preserved but functionally impaired in this group, potentially due to reduced bioactivity or molecular alterations that limit its ability to stimulate cortisol production. Conversely, the ACTH-depleted group exhibited a moderate positive correlation, indicating that although ACTH levels were reduced, its physiological activity remained relatively intact. These findings underscore the limitations of relying solely on ACTH levels when diagnosing IAD, particularly in patients with preserved-range ACTH and inadequate cortisol secretion.

These individuals, categorized as the ACTH-preserved group, showed markedly reduced serum cortisol levels and poor responsiveness to CRH stimulation. Such findings indicate a failure to produce ACTH with normal biological structure and function, reflecting compromised integrity of the hypothalamic-pituitary-adrenal axis. Although ACTH concentrations significantly differed between groups, cortisol levels remained similarly suppressed, supporting the hypothesis that circulating ACTH in the preserved group lacks biological activity and cannot stimulate adrenal steroidogenesis effectively. The blunted ACTH response to CRH stimulation observed in both groups suggests that damage to corticotrophs in the anterior pituitary is a common pathological feature in ICI-IAD, regardless of baseline ACTH levels. Additionally, this result highlights the potential utility of CRH stimulation testing in confirming the diagnosis of ICI-IAD when biochemical findings are ambiguous.

The Synacthen test confirmed that adrenal reserve was profoundly impaired in both ACTH-preserved and ACTH-depleted patients, reinforcing that normal-range ACTH values do not exclude clinically relevant adrenal insufficiency. In addition to this, CRH stimulation testing provided complementary information by directly assessing corticotroph responsiveness. Both preserved and depleted groups exhibited blunted ACTH responses, indicating pituitary dysfunction even when basal ACTH appeared normal. Thus, while the Synacthen test is indispensable for confirming adrenal insufficiency, the CRH test provides valuable mechanistic insight into the level of hypothalamic–pituitary axis dysfunction. The inclusion of both tests in our study not only reinforces our main hypothesis but also enhances the understanding of ICI-IAD from both diagnostic and pathophysiological perspectives. The parallel assessment of CRH and Synacthen responses further highlighted the concordance between pituitary dysfunction and adrenal unresponsiveness, supporting the concept of unified hypothalamic-pituitary-adrenal-axis failure in ICI-IAD. Although the Synacthen test was performed in only a subset of patients, the consistent finding of impaired adrenal reserve in all tested cases provides supportive evidence for our main conclusion that basal ACTH levels, even when preserved, cannot exclude ICI-IAD. At the same time, performing Synacthen testing in all patients would have further strengthened the robustness of our findings by eliminating potential selection bias and allowing a more comprehensive comparison across the entire cohort.

The cut-off of 10 pg/mL used to classify patients into ACTH-preserved and ACTH-depleted groups was pragmatically adopted from criteria for subclinical Cushing’s syndrome, and its validity in the context of ICI-IAD has not been established. Nevertheless, sensitivity analyses using alternative thresholds (5, 7.5, and 15 pg/mL) yielded consistent results, indicating that our main conclusions were robust to the choice of cut-off. Larger studies will be required to determine the most appropriate thresholds specific to this condition.

To investigate potential structural abnormalities in ACTH, GFC was employed. ACTH from the preserved group showed peaks that eluted earlier than the standard ACTH (1–39), indicating a higher molecular weight and suggesting structural alterations. Additionally, peak ACTH values in each sample were corrected using plasma ACTH concentrations measured by the AIA system, ensuring accurate comparisons by minimizing the influence of inter-individual variability in secretion levels. This correction enabled the evaluation of actual peak concentrations independent of baseline ACTH levels. The preserved group showed significantly elevated corrected concentrations of high-molecular-weight ACTH, indicating the presence of structurally modified ACTH variants. Although ACTH levels remained within the reference range, insufficient cortisol secretion and early elution profiles observed through chromatography pointed to physicochemical abnormalities in the ACTH molecule. These abnormalities may represent pro-ACTH or proopiomelanocortin (POMC) fragments produced through incomplete prohormone processing, or ACTH altered by defective post-translational modifications, including glycosylation or phosphorylation (20–22). Proper processing of POMC in the anterior pituitary is mainly mediated by prohormone convertase PC1/3 (23), and ICI-induced local inflammation or autoimmunity (24) may impair its enzymatic activity (25), leading to accumulation of aberrant ACTH. The biological activity of ACTH is critically dependent on its tertiary structure and binding affinity to the melanocortin 2 receptor (MC2R) (26, 27), and such modifications may impair its function. These findings underscore the importance of evaluating both the “quantity” and “quality” of ACTH in understanding the pathophysiology of ICI-IAD and highlight the need for further structural and functional analyses of the ACTH molecule. In addition, beyond adrenal cortisol regulation, ACTH and other POMC-derived peptides have pleiotropic effects on diverse tissues such as melanocytes, adipocytes, and immune cells (28), suggesting that ICI-induced alterations of ACTH biology may have broader systemic implications.

To support this hypothesis, adrenal steroid profiles were compared between the groups. Downstream adrenal steroids, including pregnenolone, progesterone, and cortisol, showed similar suppression across both groups without significant differences. This consistent suppression aligns with the clinical observation of impaired adrenal responsiveness to ACTH and suggests a loss of physiological bioactivity in the ACTH-preserved group.

Furthermore, in one representative case, serial GFC was performed at three time points—before ICI initiation, during ICI treatment before the onset of adrenal insufficiency, and after the development of ICI−IAD—revealing a progressive increase in high-molecular-weight ACTH. As shown in **Supplementary Figure S2**, this temporal trend supports the hypothesis that ICI exposure may promote the emergence of structurally altered, potentially bio−inactive ACTH forms. Immune−mediated mechanisms, including ectopic POMC expression and autoimmunity against corticotrophs, have been implicated in ICI−induced hypophysitis and may underlie these molecular changes (29, 30). Paraneoplastic−like processes—where tumor-derived ectopic POMC expression triggers auto−reactivity to corticotrophs—have been described, particularly in patients treated with anti–PD−1/PD−L1 agents (30, 31).

These findings collectively indicate a novel pathophysiological mechanism in ICI-IAD involving irreversible damage to ACTH-secreting cells and secretion of structurally altered, biologically inactive ACTH. Exclusive reliance on plasma ACTH concentrations to rule out adrenal insufficiency may lead to misdiagnosis. Supplementary assessments such as CRH stimulation testing, detailed steroid profiling, and ultimately ACTH structural analysis may enhance diagnostic accuracy. With the emergence of next-generation ICIs, including lymphocyte-activation gene 3 (LAG-3) blocking antibodies such as relatlimab, the clinical relevance of accurately diagnosing ICI-IAD is expected to grow. Our findings may help improve diagnostic strategies in the evolving landscape of cancer immunotherapy.

A notable strength of our study is the incorporation of a prospectively followed cohort with consecutive enrollment and standardized collection of paired plasma and serum alongside harmonized clinical data capture. This approach minimizes selection and information bias, reduces pre-analytical variability, and enables integrated analyses across assays (steroid profiling, ACTH bioactivity, and structural characterization) with alignment to dynamic testing where available. Notably, this integrated approach enhanced the robustness of our findings and ensured reproducibility when combining retrospective and prospective data. The availability of paired serum and plasma samples under standardized conditions also provides a valuable platform for future mechanistic studies, including structural characterization and functional evaluation of ACTH variants.

From a clinical perspective, careful interpretation of ACTH values is essential, as basal ACTH levels alone are not sufficient to exclude ICI-IAD. The initial assessment should include clinical symptoms together with basal ACTH and cortisol levels. When these parameters are clearly abnormal, the diagnosis is usually straightforward. However, when findings are not typical—for example, when ACTH appears inappropriately elevated relative to cortisol—dynamic testing can provide valuable additional information. This study demonstrated the usefulness of both the short Synacthen test and CRH stimulation, which provide complementary insights into adrenal reserve and pituitary function. Incorporating these tests into diagnostic workflows, particularly when basal findings are inconclusive, may improve the timely and accurate recognition of ICI-IAD.

This study has certain limitations. First and most notably, the ACTH-preserved group included only seven patients, which limits statistical power for group comparisons and correlation analyses. The significance of some findings may therefore have been influenced by the small sample size. Second, the observations were derived from a single-center, small cohort, and caution is warranted when generalizing the results to populations with different backgrounds. Accordingly, validation in larger, multicenter studies will be necessary. Third, although the short Synacthen test was incorporated, it could only be performed in 24 patients due to institutional protocols, which may have introduced selection bias. Finally, both the GFC and steroid profiling analyses were restricted to 10 patients with sufficient stored samples, limiting the generalizability of these specific results, although consistent patterns were observed across ACTH-preserved and ACTH-depleted groups. The prospective phase of our study is still ongoing, and future expansion of these analyses in additional cases will be important to strengthen these results.

Another important limitation involves the indirect assessment of ACTH bioactivity. The inference of biological inactivity was based on persistently low cortisol levels, blunted CRH responses, suppressed steroidogenesis, detection of high-molecular-weight ACTH isoforms, and a lack of correlation with downstream steroid levels. Direct confirmation of ACTH bioactivity will require validation through functional bioassays, particularly those employing adrenal cells exposed to high-molecular-weight ACTH isoforms. Furthermore, the interpretation that the high-molecular-weight ACTH detected in the ACTH-preserved group reflects abnormal POMC processing or post-translational modification remains provisional. To clarify this, further experimental validation will be important. Future work will focus on structural characterization of circulating ACTH species using LC-MS/MS peptide mapping to identify potential modifications or aberrant peptide processing, as well as functional bioassays to directly assess their biological activity. These approaches, facilitated by ongoing prospective sample collection, are expected to help determine both the molecular basis and the physiological relevance of these ACTH variants.

In conclusion, the findings indicate that a subset of patients with ICI-IAD may present with preserved ACTH concentrations despite biochemical evidence of adrenal insufficiency. This phenomenon likely results from the secretion of structurally abnormal, biologically inactive ACTH. A thorough endocrine evaluation—beyond isolated hormone measurements—plays a critical role in accurate diagnosis. Normal ACTH should not be interpreted as definitive evidence against adrenal insufficiency during ICI therapy. Careful clinical assessment remains essential to ensure timely recognition and management. Future studies are warranted to clarify the molecular characteristics and bioactivity of ACTH in these cases. Functional assays, LC-MS/MS-based profiling, and longitudinal prospective studies may provide deeper insights into the diagnostic value of ACTH dynamics and aid in establishing more refined criteria for ICI-IAD.

## Data Availability

The raw data supporting the conclusions of this article will be made available by the authors, without undue reservation.

## References

[1] Shiravand Y Khodadadi F Kashani SMA Hosseini-Fard SR Hosseini S Sadeghirad H. Immune checkpoint inhibitors in cancer therapy. Curr Oncol. (2022) 29:3044–60. doi: 10.3390/curroncol29050247, PMID: 35621637 PMC9139602

[2] Miller SR Schipper M Fritsche LG Jiang R Strohbehn G Ötleş E. Pan-cancer survival impact of immune checkpoint inhibitors in a national healthcare system. Cancer Med. (2024) 13:e70379. doi: 10.1002/cam4.70379, PMID: 39508134 PMC11541111

[3] Sun Q Hong Z Zhang C Wang L Han Z Ma D. Immune checkpoint therapy for solid tumours: clinical dilemmas and future trends. Signal Transduct Target Ther. (2023) 8:320. doi: 10.1038/s41392-023-01522-4, PMID: 37635168 PMC10460796

[4] Qinan Y Liuyun W Lizhu H Xingyue Z Rongsheng T Lian L. Immune-related adverse events of immune checkpoint inhibitors: a review. Front Immunol. (2023) 14:1167975. doi: 10.3389/fimmu.2023.1167975, PMID: 37304306 PMC10247998

[5] Eileen LC Renee L Kiandokht K Katie H Meghan B M Tariq B. Consensus disease definitions for ophthalmic immune-related adverse events of immune checkpoint inhibitors. J Immunother Cancer. (2025) 13:e011049. doi: 10.1136/jitc-2024-011049, PMID: 40199607 PMC11979595

[6] Barron CC Stefanova I Cha Y Elsolh K Zereshkian A Gaafour N. Chronic immune-related adverse events in patients with cancer receiving immune checkpoint inhibitors: a systematic review. J Immunother Cancer. (2023) 11:e006500. doi: 10.1136/jitc-2022-006500, PMID: 37536939 PMC10401216

[7] Wright JJ Powers AC Johnson DB. Endocrine toxicities of immune checkpoint inhibitors. Nat Rev Endocrinol. (2021) 17:389–99. doi: 10.1038/s41574-021-00484-3, PMID: 33875857 PMC8769055

[8] Poto R Troiani T Criscuolo G Marone G Ciardiello F Tocchetti CG. Holistic approach to immune checkpoint inhibitor-related adverse events. Front Immunol. (2022) 13:804597. doi: 10.3389/fimmu.2022.804597, PMID: 35432346 PMC9005797

[9] Kamitani F Nishioka Y Koizumi M Nakajima H Kurematsu Y Okada S. Immune checkpoint inhibitor-related type 1 diabetes incidence, risk, and survival association. J Diabetes Investig. (2025) 16:334–42. doi: 10.1111/jdi.14362, PMID: 39569589 PMC11786175

[10] Brahmer JR Lacchetti C Schneider BJ Atkins MB Brassil KJ Caterino JM. Management of immune-related adverse events in patients treated with immune checkpoint inhibitor therapy: American Society of Clinical Oncology Clinical Practice Guideline. J Clin Oncol. (2018) 36:1714–68. doi: 10.1200/jco.2017.77.6385, PMID: 29442540 PMC6481621

[11] Network NCC. NCCN clinical practice guidelines in oncology: management of immunotherapy-related toxicities (Version 2.2024). PA: Plymouth Meeting (2024).

[12] Haanen JBAG Carbonnel F Robert C Kerr KM Peters S Larkin J. Management of toxicities from immunotherapy: ESMO Clinical Practice Guidelines for diagnosis, treatment and follow-up. Ann Oncol. (2017) 28:iv119–42. doi: 10.1093/annonc/mdx225, PMID: 28881921

[13] Yudai H Nobumasa O Yuhki S Koda R Yoneoka Y Takada T. Isolated adrenocorticotropic hormone deficiency associated with severe hyperkalemia during pembrolizumab therapy in a patient with ureteral cancer and an ileal conduit: a case report and literature review. Am J Case Rep. (2021) 22:e931639. doi: 10.12659/AJCR.931639, PMID: 34262010 PMC8297058

[14] Kanie K Iguchi G Bando H Fujita Y Odake Y Yoshida K. Two cases of atezolizumab-induced hypophysitis. J Endocr Soc. (2018) 2:91–5. doi: 10.1210/js.2017-00414, PMID: 29362727 PMC5774896

[15] Yamauchi I Taura D Hakata T Fujita H Okamoto K Ueda Y. Clinical features and thyroid dysfunction in adverse events involving the pituitary gland during PD-1 blockade therapy. Clin Endocrinol. (2021) 94:258–68. doi: 10.1111/cen.14349, PMID: 33037658

[16] Bando H Yamamoto M Urai S Motomura Y Sasaki Y Ohmachi Y. Fluctuations in plasma adrenocorticotropic hormone concentration may predict the onset of immune checkpoint inhibitor-related hypophysitis. J Immunother Cancer. (2024) 12:e008634. doi: 10.1136/jitc-2023-008634, PMID: 38418395 PMC10910626

[17] Yanase T Oki Y Katabami T Otsuki M Kageyama K Tanaka T. New diagnostic criteria of adrenal subclinical Cushing’s syndrome: opinion from the Japan Endocrine Society. Endocr J. (2018) 65:383–93. doi: 10.1507/endocrj.ej17-0456, PMID: 29576599

[18] Nieman LK Biller BMK Findling JW Newell-Price J Savage MO Stewart PM. The diagnosis of Cushing’s syndrome: an endocrine society clinical practice guideline. J Clin Endocrinol Metab. (2008) 93:1526–40. doi: 10.1210/jc.2008-0125, PMID: 18334580 PMC2386281

[19] Chamorro-Pareja N Faje AT Miller KK. Pituitary complications of checkpoint inhibitor use. Endocrinology. (2024) 165:bqae084. doi: 10.1210/endocr/bqae084, PMID: 39001874

[20] Simó R Hernández C Chacón P Martí R Mesa J. Effect of insulin administration on serum lipoprotein(a) and its phenotypes in new-onset IDDM patients. Diabetes Care. (1998) 21:866–7. doi: 10.2337/diacare.21.5.866, PMID: 9589257

[21] Takahashi A Mizusawa K. Posttranslational modifications of proopiomelanocortin in vertebrates and their biological significance. Front Endocrinol. (2013) 4:143. doi: 10.3389/fendo.2013.00143, PMID: 24146662 PMC3797980

[22] Cawley NX Li Z Loh YP. 60 YEARS OF POMC: biosynthesis, trafficking, and secretion of pro-opiomelanocortin-derived peptides. J Mol Endocrinol. (2016) 56:T77–97. doi: 10.1530/jme-15-0323, PMID: 26880796 PMC4899099

[23] Bicknell AB. The tissue-specific processing of pro-opiomelanocortin. J Neuroendocrinol. (2008) 20:692–9. doi: 10.1111/j.1365-2826.2008.01709.x, PMID: 18601691

[24] Chiloiro S Russo F Tartaglione T Capoluongo ED. Molecular and genetic immune biomarkers of primary and immune-therapy induced hypophysitis: from laboratories to the clinical practice. J Pers Med. (2021) 11:1026. doi: 10.3390/jpm11101026, PMID: 34683167 PMC8537090

[25] Stanke-Labesque F Gautier-Veyret E Chhun S Guilhaumou R French Society of Pharmacology and Therapeutics. Impact of infectious and inflammatory disease on cytochrome P450–mediated drug metabolism and pharmacokinetics. Pharmacol Ther. (2020) 215:107627. doi: 10.1016/j.pharmthera.2020.107627, PMID: 32659304 PMC7351663

[26] Fridmanis D Roga A Klovins J. ACTH receptor (MC2R) specificity: what do we know about underlying molecular mechanisms? Front Endocrinol. (2017) 8:13. doi: 10.3389/fendo.2017.00013, PMID: 28220105 PMC5292628

[27] Luo P Feng W Ma S Dai A Wu K Chen X. Structural basis of signaling regulation of the human melanocortin-2 receptor by MRAP1. Cell Res. (2023) 33:46–54. doi: 10.1038/s41422-022-00751-6, PMID: 36588120 PMC9810661

[28] Hasenmajer V Bonaventura I Minnetti M Sada V Sbardella E Isidori AM. Non-canonical effects of ACTH: insights into adrenal insufficiency. Front Endocrinol (Lausanne). (2021) 12:701263. doi: 10.3389/fendo.2021.701263, PMID: 34489864 PMC8416901

[29] Kanie K Iguchi G Bando H Urai S Shichi H Fujita Y. Mechanistic insights into immune checkpoint inhibitor-related hypophysitis: a form of paraneoplastic syndrome. Cancer Immunol Immunother. (2021) 70:3669–77. doi: 10.1007/s00262-021-02955-y, PMID: 33977343 PMC8571153

[30] Di Stasi V La Sala D Cozzi R Scavuzzo F De Geronimo V Poggi M. Immunotherapy-related hypophysitis: a narrative review. Cancers. (2025) 17:436. doi: 10.3390/cancers17030436, PMID: 39941803 PMC11815778

[31] Urai S Watanabe M Bando H Motomura Y Yamamoto M Tachihara M. Paraneoplastic isolated adrenocorticotropic hormone deficiency revealed after immune checkpoint inhibitors therapy: new insights into anti-corticotroph antibody. Front Immunol. (2023) 14:1284301. doi: 10.3389/fimmu.2023.1284301, PMID: 38035072 PMC10682701

