# Supplementary figures and images for: Normal adrenocorticotropic hormone levels do not exclude adrenal insufficiency during immune checkpoint inhibitor therapy: evidence from clinical, steroid, and structural analyses
Source: Front Endocrinol (Lausanne). 2025 Oct 20;16:1683546. doi: 10.3389/fendo.2025.1683546 (PMC12580128; doi:10.3389/fendo.2025.1683546)

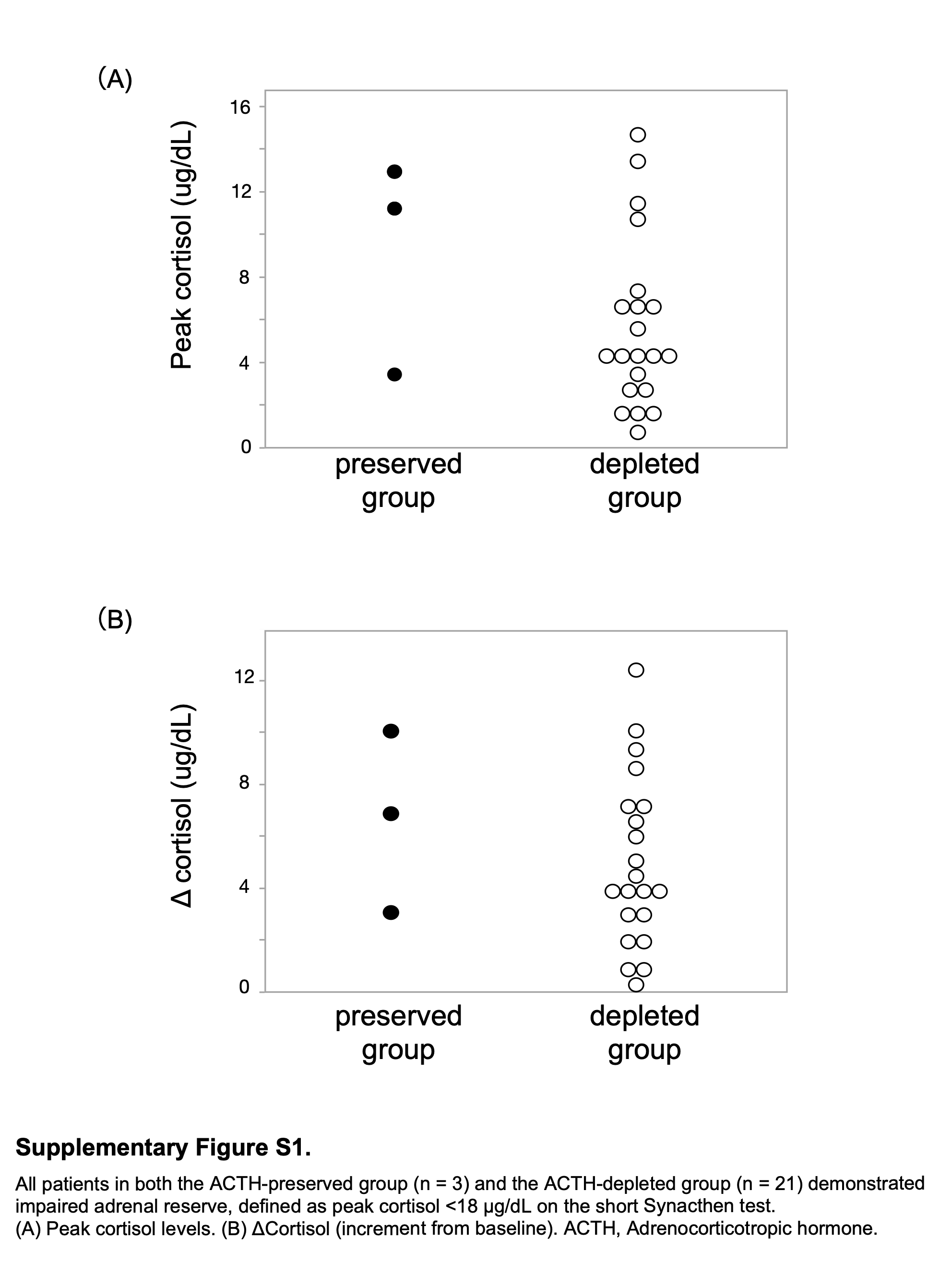

Supplement: Supplementary file 1 [file Image1.tiff]

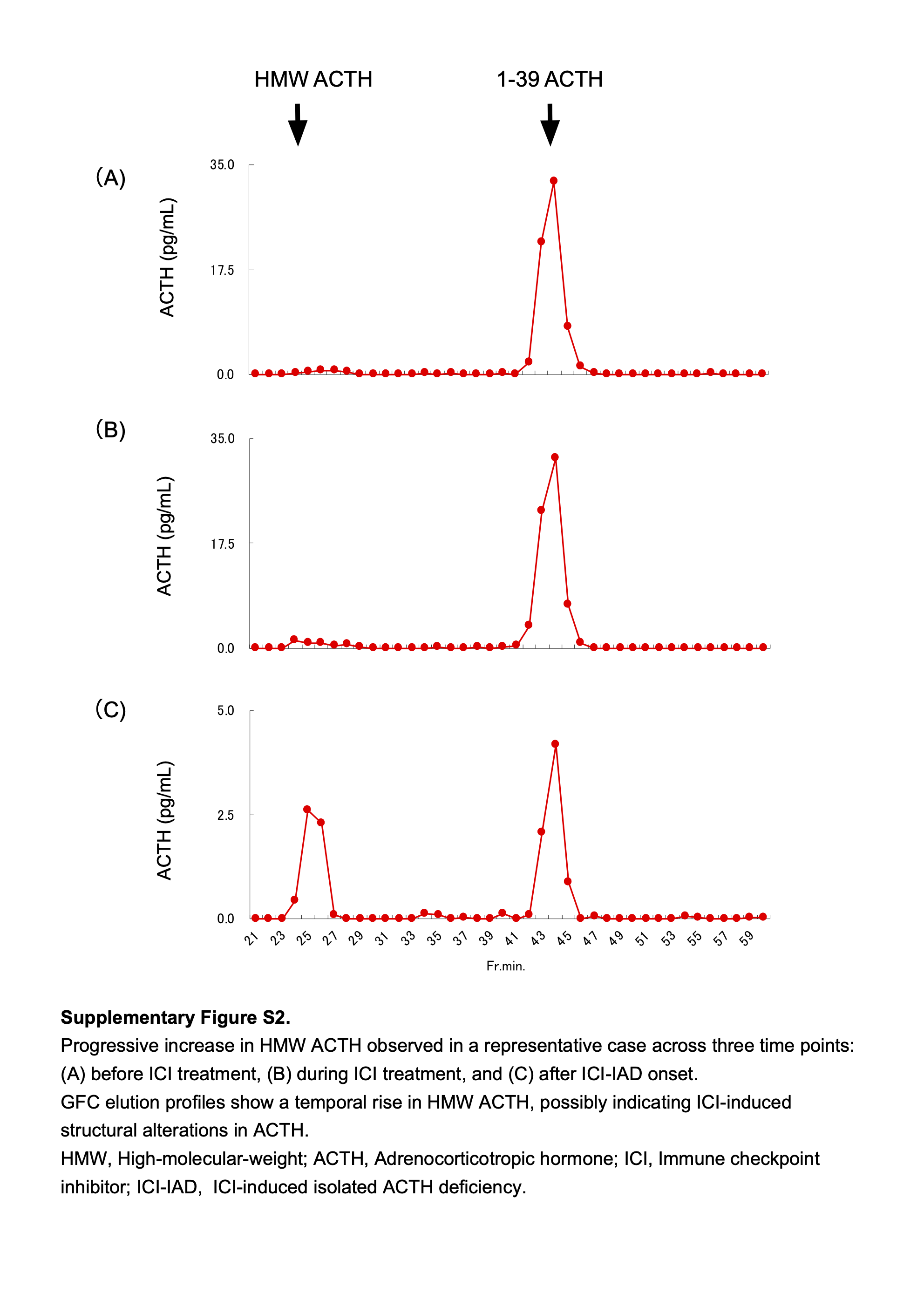

Supplement: Supplementary file 2 [file Image2.tiff]
