# Supplementary material for: Normal adrenocorticotropic hormone levels do not exclude adrenal insufficiency during immune checkpoint inhibitor therapy: evidence from clinical, steroid, and structural analyses
Source: Front Endocrinol (Lausanne). 2025 Oct 20;16:1683546. doi: 10.3389/fendo.2025.1683546 (PMC12580128; doi:10.3389/fendo.2025.1683546)
Supplement: Supplementary file 3 [file Table1.docx]

Supplementary Material

**Supplementary Table S1.** Clinical characteristics of prospectively and retrospectively enrolled patients.

|  | **retrospective group (n=25)** | **prospective group (n=24)** | ***P* value** |
| --- | --- | --- | --- |
| Age at diagnosis, median (IQR) | 65 (61–72) | 72 (70–75) | 0.01 |
| Male, n (%) | 16 (64.0) | 19 (79.2) | 0.24 |
| **Tumor type, n (%)** |  |  | 0.27 |
| Lung | 9 (36.0) | 5 (20.8) |  |
| Liver | 1 (4.0) | 8 (33.3) |  |
| Oral cavity | 4 (16.0) | 1 (4.2) |  |
| Gastric | 1 (4.0) | 3 (12.5) |  |
| Melanoma | 3 (12.0) | 1 (4.2) |  |
| Pleural mesothelioma | 2 (8.0) | 2 (8.3) |  |
| Breast | 1 (4.0) | 1 (4.2) |  |
| Ovary | 1 (4.0) | 1 (4.2) |  |
| Urothelial | 1 (4.0) | 1 (4.2) |  |
| Colon | 1 (4.0) | 0 (0) |  |
| Kidney | 0 (0) | 1 (4.2) |  |
| Unknown primary | 1 (4.0) | 0 (0) |  |
| **ICI type, n (%)** |  |  | 0.06 |
| Anti PD-1 | 14 (56.0) | 7 (29.2) |  |
| Anti PD-L1 | 5 (20.0) | 4 (16.7) |  |
| Anti CTLA-4 | 1 (4.0) | 0 (0) |  |
| Anti PD-(L)1 + Anti CTLA-4 | 4 (16.0) | 13 (54.2) |  |
| Anti PD-1 + Anti PD-L1 | 1 (4.0) | 0 (0) |  |
| Time to develop IAD after starting ICI　months, median (IQR) | 5.8 (2.4-8.8) | 6.5 (4.2–9.2) | 0.39 |
| Number of ICI administrations until the development of IAD, median (IQR) | 5 (3–11) | 4.5 (3–7) | 0.52 |
| **Symptoms at diagnosis, n (%)** |  |  |  |
| Fatigue | 21 (84.0) | 19 (79.2) | 0.66 |
| Anorexia | 16 (64.0) | 19 (79.2) | 0.24 |
| Nausea/vomiting | 5 (20.0) | 13 (54.2) | 0.01 |
| Diarrhea | 1 (4.0) | 2 (8.3) | 0.53 |
| Fever | 5 (20.0) | 5 (20.8) | 0.94 |
| **Pituitary MRI, n (%)** |  |  | 0.27 |
| Empty sella | 1 (4.0) | 2 (8.3) |  |
| Enlargement of the pituitary gland | 0 (0) | 2 (8.3) |  |
| No remarkable change | 24 (96.0) | 20 (83.4) |  |
| **Other irAEs, n (%)** |  |  | 0.22 |
| Thyroid dysfunction | 7 (28.0) | 3 (12.5) |  |
| Liver | 1 (4.0) | 0 (0) |  |
| None | 17 (68.0) | 21 (87.5) |  |
| ACTH (pg/mL) | 4.25 (1.5–8.2) | 2.6 (1.5–7.5) | 0.55 |
| Cortisol (μg/dL) | 0.6 (0.35–1.4) | 0.6 (0.3–1.5) | 0.93 |
| WBC (/μL) | 4240 (3460–5390) | 5120 (3610–7790) | 0.07 |
| Eo (/μL) | 250 (100–505) | 480 (330–820) | 0.01 |
| Serum sodium (mmol/L) | 133 (129–138) | 136 (132–137) | 0.19 |
| Serum potassium (mmol/L) | 4.0 (3.7–4.3) | 3.9 (3.7–4.3) | 0.75 |
| Serum chloride (mmol/L) | 99 (98–105) | 102 (99–104) | 0.42 |

*P* values were calculated using the Mann-Whitney U test and Fisher’s exact test, as appropriate.

**Abbreviations:** ACTH, adrenocorticotropic hormone; ICI, immune checkpoint inhibitor; MRI, magnetic resonance imaging; WBC, white blood cell count; Eo, eosinophils.
