# Supplementary material for: Normal adrenocorticotropic hormone levels do not exclude adrenal insufficiency during immune checkpoint inhibitor therapy: evidence from clinical, steroid, and structural analyses
Source: Front Endocrinol (Lausanne). 2025 Oct 20;16:1683546. doi: 10.3389/fendo.2025.1683546 (PMC12580128; doi:10.3389/fendo.2025.1683546)
Supplement: Supplementary file 4 [file Table2.docx]

Supplementary Material

**Supplementary Table S2.** Clinical characteristics of 10 patients who underwent both LC-MS/MS based steroid profiling and GFC.

|  | **Overall (n=10)** | **Preserved group (n=3)** | **Depleted group (n=7)** | ***P* value** |
| --- | --- | --- | --- | --- |
| Age at diagnosis, median (IQR) | 72 (69–75) | 71 (70–74) | 73 (67–78) | 0.65 |
| Male, n (%) | 8 (80.0) | 2 (66.7) | 6 (85.7) | 0.50 |
| **Tumor type, n (%)** |  |  |  | 0.52 |
| Lung | 3 (30.0) | 1 (33.3) | 2 (28.6) |  |
| Liver | 4 (40.0) | 1 (33.3) | 3 (42.9) |  |
| Oral cavity | 1 (10.0) | 0 (0) | 1 (14.3) |  |
| Gastric | 1 (10.0) | 1 (33.3) | 0 (0) |  |
| Breast | 1 (10.0) | 0 (0) | 1 (14.3) |  |
| **ICI type, n (%)** |  |  |  | 0.16 |
| Anti PD-1 | 3 (30.0) | 2 (66.7) | 1 (14.3) |  |
| Anti PD-L1 | 3 (30.0) | 1 (33.3) | 2 (28.6) |  |
| Anti PD-(L)1 + Anti CTLA-4 | 4 (40.0) | 0 (0) | 4 (57.1) |  |
| Time to develop IAD after starting ICI　months, median (IQR) | 6.3 (4.2–8.9) | 6.0 (5.8–13.2) | 6.3 (4.0–8.8) | 0.55 |
| Number of ICI administrations until the development of IAD, median (IQR) | 5 (3–9) | 7 (3–18) | 5 (3–9) | 0.11 |
| **Symptoms at diagnosis, n (%)** |  |  |  |  |
| Fatigue | 8 (80.0) | 2 (66.7) | 6 (85.7) | 0.50 |
| Anorexia | 7 (70.0) | 1 (33.3) | 6 (85.7) | 0.10 |
| Nausea/vomiting | 5 (50.0) | 0 (0) | 5 (71.4) | 0.04 |
| Diarrhea | 1 (10.0) | 0 (0) | 1 (14.3) | 0.50 |
| Fever | 1 (10.0) | 0 (0) | 1 (14.3) | 0.50 |
| **Pituitary MRI, n (%)** |  |  |  | 0.46 |
| Empty sella | 1 (10.0) | 0 (0) | 1 (14.3) |  |
| Enlargement of the pituitary gland | 1 (10.0) | 1 (33.3) | 0 (0) |  |
| No remarkable change | 8 (80.0) | 2 (66.7) | 6 (85.7) |  |
| **Other irAEs, n (%)** |  |  |  | 0.30 |
| Thyroid dysfunction | 2 (20.0) | 0 (0) | 2 (28.6) |  |
| None | 8 (80.0) | 3 (100) | 5 (71.4) |  |
| ACTH (pg/mL) | 3.1 (1.5–8.1) | 19.5 (12.6–30.5) | 2.5 (1.5–5.6) | <0.0001 |
| Cortisol (μg/dL) | 0.6 (0.3–1.4) | 1.4 (0.4–1.9) | 0.55 (0.3–1.33) | 0.35 |
| WBC (/μL) | 4770 (3650–6075) | 4540 (3690–5560) | 4820 (3590–6225) | 0.43 |
| Eo (/μL) | 360 (205–640) | 410 (320–640) | 330 (200–668) | 0.97 |
| Serum sodium (mmol/L) | 135 (131–138) | 133 (129–139) | 135 (131–137) | 0.92 |
| Serum potassium (mmol/L) | 4 (3.7–4.3) | 3.8 (3.7–4.1) | 4 (3.7–4.3) | 0.49 |
| Serum chloride (mmol/L) | 100 (98–104) | 103 (97–106) | 100 (98–104) | 0.46 |

*P* values were calculated using the Mann-Whitney U test and Fisher’s exact test, as appropriate.

**Abbreviations:** ACTH, adrenocorticotropic hormone; ICI, immune checkpoint inhibitor; MRI, magnetic resonance imaging; WBC, white blood cell count; Eo, eosinophils.
